# Mistaken assumptions drive new Six Sigma model off the road

**DOI:** 10.11613/BM.2019.010903

**Published:** 2018-12-15

**Authors:** Sten Westgard, Hassan Bayat, James O Westgard

**Affiliations:** 1 Westgard QC, Madison, USA; 2 Immunogenetics Research Center, Mazandaran University of Medical Sciences, Sari, Iran; 3 University of Wisconsin School of Medicine and Public Health, Madison, USA

**Keywords:** Sigma metrics, Six Sigma, allowable total error

## Abstract

Oosterhuis and Coskun recently proposed a new model for applying the Six Sigma concept to laboratory measurement processes. In criticizing the conventional Six Sigma model, the authors misinterpret the industrial basis for Six Sigma and mixup the Six Sigma “counting methodology” with the “variation methodology”, thus many later attributions, conclusions, and recommendations are also mistaken. Although the authors attempt to justify the new model based on industrial principles, they ignore the fundamental relationship between Six Sigma and the process capability indices. The proposed model, the Sigma Metric is calculated as the ratio CV_I_/CV_A_, where CV_I_ is individual biological variation and CV_A_ is the observed analytical imprecision. This new metric does not take bias into account, which is a major limitation for application to laboratory testing processes. Thus, the new model does not provide a valid assessment of method performance, nor a practical methodology for selecting or designing statistical quality control procedures.

Oosterhuis and Coskun recently proposed a new model for applying the Six Sigma concept to laboratory measurement processes (*1*). Unfortunately, the authors misinterpret the industrial basis for Six Sigma and misuse the “counting” methodology instead of the “variation” methodology early in the development of the model, thus many later attributions, conclusions, and recommendations are also mistaken.

Although the authors attempt to justify the new model based on industrial principles, they ignore the fundamental relationship between Six Sigma and the process capability indices Cp and Cpk. Such indices were widely used in industry prior to the formalization of Six Sigma in the 1990s and provide the proper framework for understanding the development of Six Sigma (*2*). Cp is a performance index that is calculated from the difference between the upper and lower tolerance limits and the variation observed for the production process:

Cp = (TLu – TLl)/6SD (Eq. 1).

where TL_u_ is the upper tolerance limit, TL_l_ the lower tolerance limit, and SD is the standard deviation for the observed process variation, as illustrated in Figure 1 (A). The distribution of measurements is assumed to be Gaussian (normal) around the target value (TV). For laboratory testing processes, it is common to express the tolerance limits in terms of an allowable total error (ATE, TEa), or pTE, the abbreviation chosen by the authors for the permissible total error. Give that TL_u_ = TV + pTE, and TL_l_ = TV – pTE,

Cp = [(TV + pTE) – (TV – pTE)]/6SD = 2pTE/6SD = pTE/3SD (Eq. 2).

**Figure 1:**
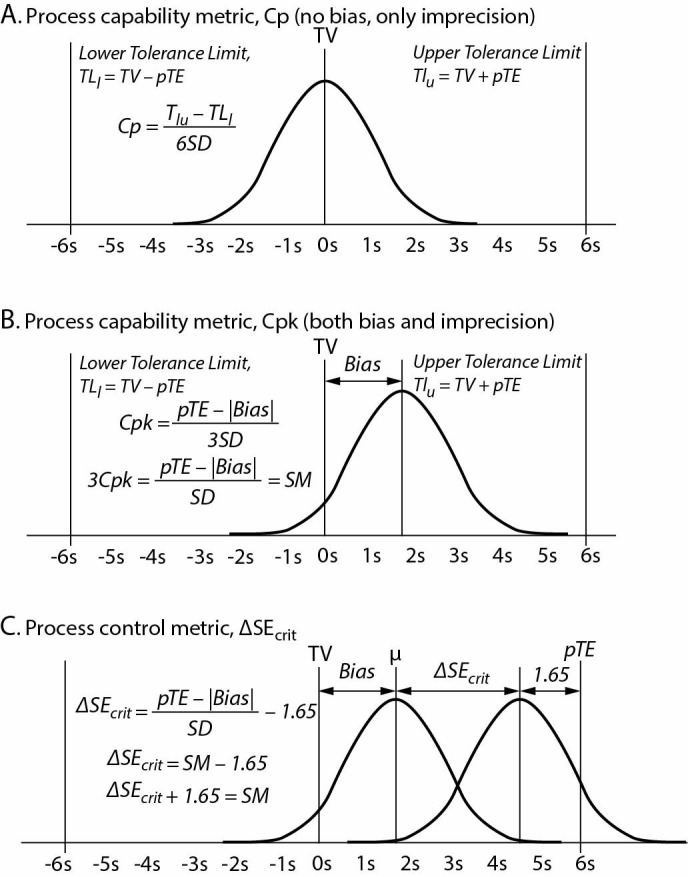
Relation of Sigma Metric (SM) to industrial process capability indices (Cp, Cpk) and process control metric (ΔSE_crit_) for SQC selection and design. ΔSEcrit - critical systematic error. TL - tolerance limit. TV - target value. Μ - observed mean. pTE - permissible total error. Bias - observed trueness. SD - observed imprecision.

Ideally, the process should operate with a Cp of 2.0, which means that ± 6 SDs or a total of 12 SDs of process variation should fit between the tolerance limits. In industry, a minimum Cp of 1.0 is considered essential for routine operation and a Cp of 1.33 is preferred. Knowledge of this relationship led to recommendations in 1990 for changing process acceptance criteria from 2SD < pTE to 4SD < pTE as a minimum and recommending further improvements to 5SD-6SD<pTE for critical medical applications (*3*). These recommendations were made long before the Six Sigma methodology was first formally applied to laboratory processes (*4*).

A limitation of Cp is that it assumes the process is centered on the TV, therefore it cannot account for any shift that might occur. Another capability index, Cpk, takes “centerness” into account and therefore provides a better metric for assessing performance of a laboratory testing process (*5*):

Cpk = min [(μ – TLl)/ 3SD, (TLu – μ)/3SD] (Eq. 3).

where μ represents the mean observed for the distribution. As shown in Figure 1 (B), “centerness” is characterized by the bias of a testing process, which is the difference between TV and μ. Bias causes a systematic shift of the measurement distribution, moving it closer to one of the tolerance limits. Under this condition, process performance is best described as the minimum value, *i.e.* [(pTE – Bias)/SD] or [(Bias – pTE)/SD]. This can also be expressed as (pTE - |Bias|), where |Bias| is the absolute value of the bias.

Cpk = (pTE - |Bias|)/3SD (Eq. 4).

Or

3Cpk = (pTE - |Bias|)/ SD = Sigma Metric (Eq. 5).

Thus, the conventional calculation of a Sigma Metric is directly related to the traditional industrial process capability index Cpk. The minimum acceptable Cpk of 1.0 is equivalent to SM = 3.0, a Cpk of 1.33 that is recommended to achieve a more controllable process corresponds to SM = 4.0, and the goal for excellent performance is a Cpk of 2.0, which corresponds to SM = 6.0 for world class quality.

Oosterhuis and Coskun state that the “*pTE – Bias term does not reflect the tolerance limit concept used in industry*”. The “Observed bias” in this context comes from the industrial concept and refers to the lack of “centerness” in Cpk, not the TE model. They misunderstand that the observed method bias is subtracted from the tolerance limit pTE because it narrows the region for acceptable performance. Bias accounts for the lack of “centerness” of the production distribution and is completely consistent with the industrial concept of Cpk, not a *“clear contradiction with the Six Sigma concept”* as claimed by the authors.

Another mistake is that the authors mix-up the Six Sigma “counting methodology” with the “variation methodology”. The counting methodology is used when inspecting products to identify defects, whereas the variation methodology is employed when process variation can be measured directly, which is the case for laboratory testing processes where regulation and accreditation guidelines actually require the laboratory to verify the precision and bias of their testing processes. The counting methodology employs a table based on the normal distribution to convert the observed number of defects expressed as DPMO (defects *per* million opportunities) to a sigma metric. As part of the counting methodology, it has been assumed that process drift equivalent to systematic errors of the magnitude 1.5 times the SD of the process may occur and go undetected. Therefore, the conversion table called “long-term Sigma” builds that shift into the numbers. Another table, called short-term Sigma, does not include that shift and is consistent with the variation methodology. (See Bayat for a detailed discussion of short-term and long-term sigmas (*6*).) The problem for the authors is their interpretation that *“in the model used in laboratory medicine, in addition to the 1.5 SD shift, the measured bias is also included…”.* That is not correct. The conventional laboratory SM model is based on variation, not counting, therefore it does not assume a 1.5 SD shift. Instead, the size of a medically important shift is calculated to guide the selection and design of statistical quality control (SQC) procedures and optimize the detection of medically important errors (*7*). This SQC selection or design metric is called the critical systematic error, ΔSE_crit_, and represents the size of the systematic error that must be detected to maintain the quality of the production process, as shown in Figure 1 (C):

ΔSEcrit = [(pTE - |Bias|)/SD] - 1.65 (Eq. 6),

where 1.65 is a z-value that defines a maximum 5% risk of reporting erroneous test results when a critical systematic shift occurs (*8*). Statistical quality control performance is then assessed from power function graphs, as shown in Figure 2, to determine the probability for error detection (P_ed_) for this critical shift and the probability of false rejection (P_fr_) for stable operating conditions (without this shift). Observe that the upper x-axis in Figure 2 represents a sigma scale and the lower y-axis the scale for the size of the critical SE, based on the relationship:

ΔSEcrit = SM - 1.65 (Eq. 7),

**Figure 2:**
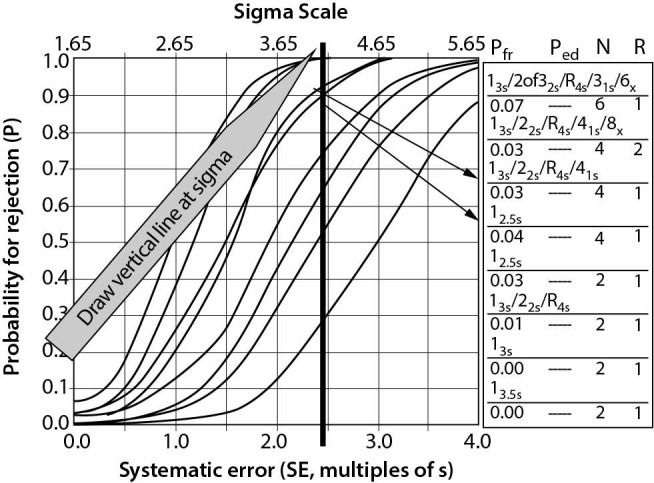
Quality planning tool for selection/design of SQC procedures having 2 levels of controls. The probability for rejection is plotted on y-axis versus the size of systematic error on bottom x-axis and the sigma-metric on the top x-axis. In the key at the right, the different power curves correspond, top to bottom, to the list of control rules, the probability for false rejection (Pfr), total number of control rules (N), and number of runs (R) over which the rules are applied. This chart was produced by the EZ Rules3 computer program. Vertical line represents examination procedure with observed sigma-metric of 4.0.

or

ΔSEcrit +1.65 = SM (Eq. 8).

Therefore, the Sigma-Metric (SM) can provide guidance for the selection and design of SQC procedures, as well as a metric for assessing the quality of performance for a testing process.

In addition to these major mistakes in the development of the new model, they further confuse the Six Sigma performance assessment model with a different goal-setting model for pTE, then combine the two models and make erroneous attributions based on the new model. The result is that the SM is calculated as the ratio CV_I_/CV_A_, where CV_I_ is the tolerance limit stated as an imprecision goal based on individual biological variation and CV_A_ is the observed analytical imprecision. This new metric does not take bias into account, which is a major limitation for application to laboratory testing processes. Furthermore, this model ignores other approaches for defining tolerance limits that are commonly employed, *e.g.*, the use of acceptable performance limits in proficiency testing and external quality assessment schemes. Thus, the new model does not provide a valid assessment of method performance, nor a practical methodology for selecting or designing SQC procedures, while also limiting the application of widely accepted test acceptability criteria that have been defined for pTE.
